# Urethral metastasis from non-seminomatous germ cell tumor: a case report

**DOI:** 10.1186/1752-1947-5-12

**Published:** 2011-01-17

**Authors:** Vijay Agarwal, Tze Wah, Sameer Chilka, Johnathan Joffe, Dan Stark

**Affiliations:** 1 St James's Institute of Oncology, Department of Medical Oncology, Leeds, UK; 2 St James's Institute of Oncology, Department of Radiology, Leeds, UK; 3 St James's Institute of Oncology, Department of Pathology, Leeds, UK

## Abstract

**Introduction:**

We present a case of nonseminomatous germ cell tumor of the testes with acute urinary retention secondary to urethral metastasis. This presentation, and similar cases of urethral metastasis from this tumor, have not been reported previously.

**Case presentation:**

A 35-year-old Caucasian man presented to hospital with a history of acute urinary retention. On examination he was found to have right testicular enlargement with raised β-human chorionic gonadotrophin, serum α-fetoprotein and lactate dehydrogenase levels. He underwent radical left inguinal orchidectomy and histology confirmed a nonseminomatous germ cell tumor of the testes. Cystoscopy carried out due to urinary retention showed penile metastasis and the biopsy confirmed metastatic malignant undifferentiated teratoma. Staging computed tomography scan and magnetic resonance imaging of the pelvis showed pulmonary, pelvic nodal, ischial and penile metastasis. The diagnosis of the International Germ Cell Cancer Collaborative Group of poor prognosis metastatic nonseminomatous germ cell tumor was made, following which he received four cycles of bleomycin, etoposide and cisplatin chemotherapy with curative intent. He had a complete marker and an excellent radiological response. He is currently under follow up.

**Conclusion:**

The unusual presentation of lymphovascular spread in this case of nonseminomatous germ cell tumor highlights the need to include routine pelvic imaging in the assessment and follow up of testicular cancer.

## Introduction

We report the case of a Caucasian man who presented with acute urinary retention and was found to have urethral metastasis from a nonseminomatous germ cell tumor. Urethral metastasis from a nonseminomatous germ cell tumor is very unusual, as it does not lie in the lymphatic drainage pathway of the testis.

## Case presentation

A 35-year-old Caucasian man presented to his local general hospital emergency department with a history of acute urinary retention. He had a background history of grade 1 astrocytoma in the posterior fossa at the age of six that was resected in 1979, and required re-excision in 1982 followed by radiotherapy resulting in residual hydrocephalus, epilepsy and mild learning difficulties. There was no history of testicular maldescent, orchidopexy or other inguino-scrotal surgery. On examination he was found to have no new neurological deficits, but right testicular enlargement was noted. An ultrasound study confirmed a 9×8 cm heterogeneous mass replacing the body of the right testis. His serum β-human chorionic gonadotrophin level was 5047 U/L (normal range 0 to 5 U/L), serum α-fetoprotein level was 7413 kU/L (normal range 3 to 8 kU/L) and lactate dehydrogenase level was 1452 IU/L (normal range 230 to 460 IU/L). His liver and renal biochemistry was normal.

**Figure 1:**
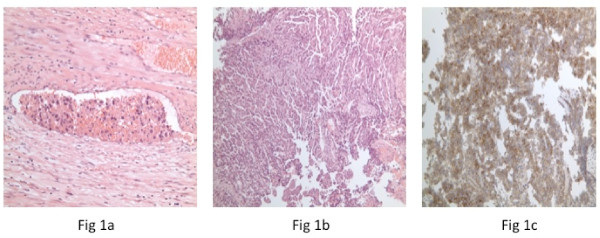
**Histology:** (a) Testicular tumor demonstrating vascular invasion by undifferentiated teratoma. (b) Urethral tumor demonstrating undifferentiated teratoma. (c) Urethral tumor demonstrating positive staining for CD30 in immunohistochemistry.

A urethral catheter was placed, and drained normal urine. He underwent a radical left inguinal orchidectomy five days later. Histology indicated a non-seminomatous germ cell tumor, with predominantly yolk sac tumor and small amounts of undifferentiated teratoma. The overall histological diagnosis was malignant teratoma intermediate (British Testicular Tumour Panel (BTTP) classification) or embryonal carcinoma (World Health Organization (WHO) classification). Lymphovascular involvement was noted, with widespread blood vessel involvement seen (Figure 1a). The spermatic cord was not involved (pT2 classification).

**Figure 2:**
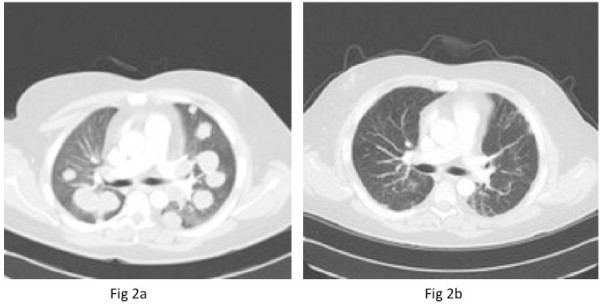
**CT Scans:** (a) Pre-chemotherapy computed tomography (CT) scan of the thorax demonstrating bilateral widespread pulmonary metastasis. (b) Post-chemotherapy CT scan of the thorax demonstrating resolution of the pulmonary metastasis.

**Figure 3:**
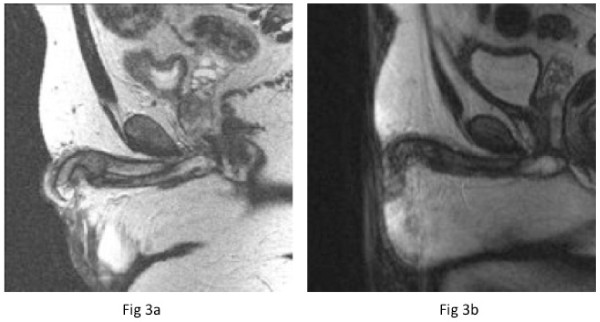
**MRI Scans:** (a) Pre-chemotherapy T2 sagittal thin penile MRI slices demonstrating the penile metastasis within the corpora. (b) Post-chemotherapy T2 sagittal thin penile MRI slices showing the resolving penile lesion.

A staging computed tomography (CT) scan revealed extensive disease within the thorax with multiple bilateral pulmonary metastases measuring up to 3 cm in both lungs (Figure 2a). In addition there was bulky mediastinal adenopathy with nodes measuring up to 3.8 cm in the prevascular space and also abnormal adenopathy was noted in both pulmonary hilum, measuring 2.6 cm versus 2.1 cm on the right and left, respectively. Two prominent interaortocaval nodes measuring up to 7 mm, bilateral 8 mm external iliac and 7.5 mm right inguinal nodes were seen. There were no significant anatomical abnormalities of the renal tract or major vessels. Postoperatively, upon catheter removal retention of urine continued. A flexible cystoscopy examination demonstrated a penile urethral tumor, and an incision biopsy was performed. A suprapubic catheter was placed. An MRI scan of the penile lesion demonstrated bilateral inguinal nodes measuring up to 9 mm along with bilateral external iliac nodes measuring up to 8 mm and bilateral internal iliac nodes smaller than 1 cm. An 8 mm pelvic lymph node adjacent to the anterolateral aspect of the bladder was noted. There were two areas of abnormality noted in the ischium, which gave a low signal on T1 and a high signal on the short T1 inversion recovery (STIR) MRI sequences consistent with bony metastasis. Within the penile shaft, 3 cm proximal to the glans penis, a soft tissue mass was confirmed measuring 2 cm in long axis, involving both the penile corpora (Figure 3a). A technetium bone scan showed a low-grade tracer uptake in the left ischium corresponding to the site of morphological abnormality on the MRI scan.

Histology of the urethral mass confirmed a metastatic malignant undifferentiated teratoma (Figure 1b). Immunochemistry was positive for placental alkaline phosphatase and CD30 (Figure 1c). The diagnosis was of an International Germ Cell Cancer Collaborative Group (IGCCCG) poor prognosis metastatic nonseminomatous germ cell tumor. Four cycles of bleomycin, etoposide and cisplatin chemotherapy were administered without complication. Then, 12 weeks after starting chemotherapy, his suprapubic catheter was clamped and he was able to micturate per urethera. His catheter was then removed. A reassessment CT scan of the chest and abdomen (Figure 2b) four weeks after completion of chemotherapy indicated excellent response to treatment. At the site of pulmonary metastasis, there was complete resolution of the pulmonary masses with only residual ill-defined areas of nodularity and lacunae seen. There was also a reduction in the size of all retroperitoneal and pelvic lymph nodes to less than 5 mm. His serum α-fetoprotein level fell to normal by week one post-chemotherapy with a plasma half life of six days, his serum β-human chorionic gonadotrophin level normalized prior to starting chemotherapy and his lactate dehydrogenase level fell to normal by week five post-chemotherapy. A repeat MRI of the pelvis six weeks after completion of chemotherapy indicated partial resolution of the penile (Figure 3a) and bony lesions. A further repeat MRI scan of the penis performed 14 weeks later confirmed further resolution of the penile metastasis, which is now a fibrotic scar-like 9 mm lesion (Figure 3b). Our patient remains in clinical, marker and radiological remission 10 months after diagnosis. His post-treatment follow-up continues.

## Discussion

Primary urethral tumors are rare, accounting for less than 1% of urological cancers in the USA [1]. Metastatic involvement of the urethra is also uncommon and is mainly a result of local spread from the surrounding organs. Germ cell tumors of the testis are the most common solid tumors in men aged between 20 and 35 years. We are not aware of any previous reports of presentation with confirmed intraluminal metastasis to the urethra, although there is a reported case of intraluminal ureteral metastasis at relapse from a recurrent testicular seminoma [2]. According to the TNM classification [3] a urethral metastasis represents stage M1b disease in the absence of evidence of local spread. Our patient's nonpulmonary visceral metastasis (urethra and bone) indicates a poor prognosis within the IGCCCG classification [3].

The spread of nonseminomatous germ cell tumors may be local, via the lymphatics or via vascular channels. Review of our patient's primary tumor revealed widespread blood vessel involvement. As the spermatic cord was not involved, it is unlikely that dissemination to the urethra represented local invasion. Seeding through urine or seminal fluid also seems unlikely. The involvement of ischium and inguinal lymph nodes is also unusual and may be attributed to lymphovascular dissemination.

In the absence of previous inguino-scrotal surgery, the usual lymphatic drainage of the right testis is through the interaortocaval nodes. Secondary lymphatic spread may cross the midline, and may occur in a retrograde fashion to the common external and inguinal nodes involving the pelvic structures. As the distal urethra drains into superficial and deep inguinal lymph nodes, it could have been involved in a retrograde manner. Spread via hematogenous dissemination is also a likely possibility.

## Conclusion

We believe this rare presentation of testicular cancer relates to atypical lymphovascular dissemination within the pelvis, with no history of surgical interventions that would predispose our patient to this route of dissemination. While the presentation in this case is highly unusual, it confirms the need to include pelvic imaging in the initial investigation of patients with testicular cancer. Furthermore, patients who have demonstrated intrapelvic dissemination of disease need to have continued pelvic imaging at every reassessment. Clinicians should consider further dedicated investigation such as penile MRI in patients presenting with urinary tract obstruction and testicular germ cell tumor.

## Consent

Written informed consent was obtained from the patient for publication of this case report and any accompanying images. A copy of the written consent is available for review by the Editor-in-Chief of this journal.

## Competing interests

The authors declare that they have no competing interests.

## Authors' contributions

VA, TW, JJ and DS were involved in conception, design, interpretation and writing of the manuscript. VA, JJ and DS were directly involved in looking after our patient. TW created and provided the radiological images. SC performed the histological examination and provided the histopathological images. All authors have been involved in critically revising the manuscript for intellectual content and they have read and approved the final version submitted.
